# SARM1-Dependent Axon Degeneration: Nucleotide Signaling, Neurodegenerative Disorders, Toxicity, and Therapeutic Opportunities

**DOI:** 10.1177/10738584231162508

**Published:** 2023-03-31

**Authors:** Helen Y. McGuinness, Weixi Gu, Yun Shi, Bostjan Kobe, Thomas Ve

**Affiliations:** 1 School of Chemistry and Molecular Biosciences, Institute for Molecular Bioscience and Australian Infectious Diseases Research Centre, University of Queensland, Saint Lucia, Australia; 2 Institute for Glycomics, Griffith University, Gold Coast, Australia

**Keywords:** NAD^+^, NMN, NaMN, NMNAT2, NADase, SARM1, axon degeneration

## Abstract

Axons are an essential component of the nervous system, and axon degeneration is an early feature of many neurodegenerative disorders. The NAD^+^ metabolome plays an essential role in regulating axonal integrity. Axonal levels of NAD^+^ and its precursor NMN are controlled in large part by the NAD^+^ synthesizing survival factor NMNAT2 and the pro-neurodegenerative NADase SARM1, whose activation triggers axon destruction. SARM1 has emerged as a promising axon-specific target for therapeutic intervention, and its function, regulation, structure, and role in neurodegenerative diseases have been extensively characterized in recent years. In this review, we first introduce the key molecular players involved in the SARM1-dependent axon degeneration program. Next, we summarize recent major advances in our understanding of how SARM1 is kept inactive in healthy neurons and how it becomes activated in injured or diseased neurons, which has involved important insights from structural biology. Finally, we discuss the role of SARM1 in neurodegenerative disorders and environmental neurotoxicity and its potential as a therapeutic target.

## Introduction

Axons are nerve fibers that extend from the bodies of nerve cells and transmit signals in response to relevant stimuli. They can be over a meter long and rely on active transport of molecules along their length, which requires considerable energy and metabolic activity. Their length and high bioenergetic demands make axons vulnerable. A range of insults can cause them to degenerate: changes in metabolism, chemicals such as cancer chemotherapy agents, neuroinflammation and necroptosis, and physical injury. Because of this vulnerability to damage, axon degeneration represents a pathologic feature of many neurodegenerative diseases that form a large part of the global disease burden, including Alzheimer disease, Parkinson disease, and amyotrophic lateral sclerosis. For these reasons, understanding the molecular mechanisms of axon degeneration has become a topic of intense research, aiming to bring new therapies to a broad range of neurodegenerative diseases.

**Figure 1.**
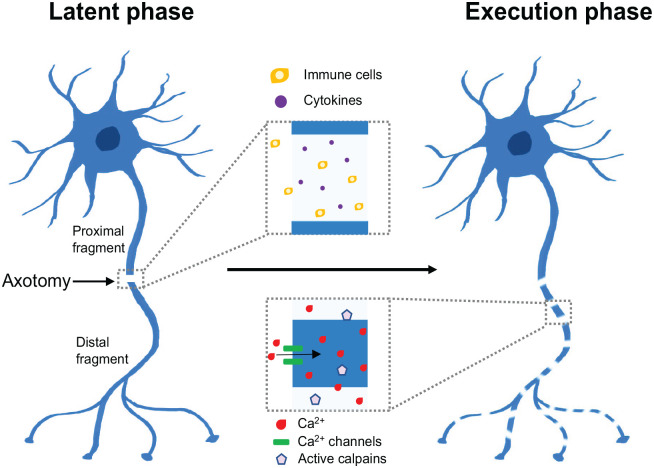
Phases of axon degeneration. Axon degeneration is characterized by two main phases: (1) a latent phase (up to 36 h), where axons remain intact but commit to degeneration and immune cells are recruited, leading to the upregulation of various cytokines; (2) an execution phase, where Ca^2+^ levels increase, calpains are activated, and axons fragment and lose their function.

A key mechanism leading to axon degeneration is termed programmed axon degeneration or Wallerian degeneration after Augustus Volney Waller’s work on frog nerves in the 1850s (Waller 1851). This process occurs in two stages. The first is a latent stage that lasts up to 36 h, where the axon commits to degeneration and nearby immune cells become activated, although the axon retains a normal appearance and continues to conduct action potentials (Figure 1). This is followed by the execution stage, where axon morphology changes and the axon loses its function and fragments. Recent years have seen tremendous advances in understanding the key players in this process, including the nucleotide NAD^+^ (nicotinamide adenine dinucleotide, oxidized form) and enzymes involved in its metabolism. We review the recent work leading to the current understanding of the process, focusing on the NAD^+^-degrading enzyme SARM1 (sterile alpha and Toll/interleukin 1 receptor motif–containing 1) and its potential as a therapeutic target.

## Key Molecular Players of Axon Degeneration

### NAD^+^

**Figure 2.**
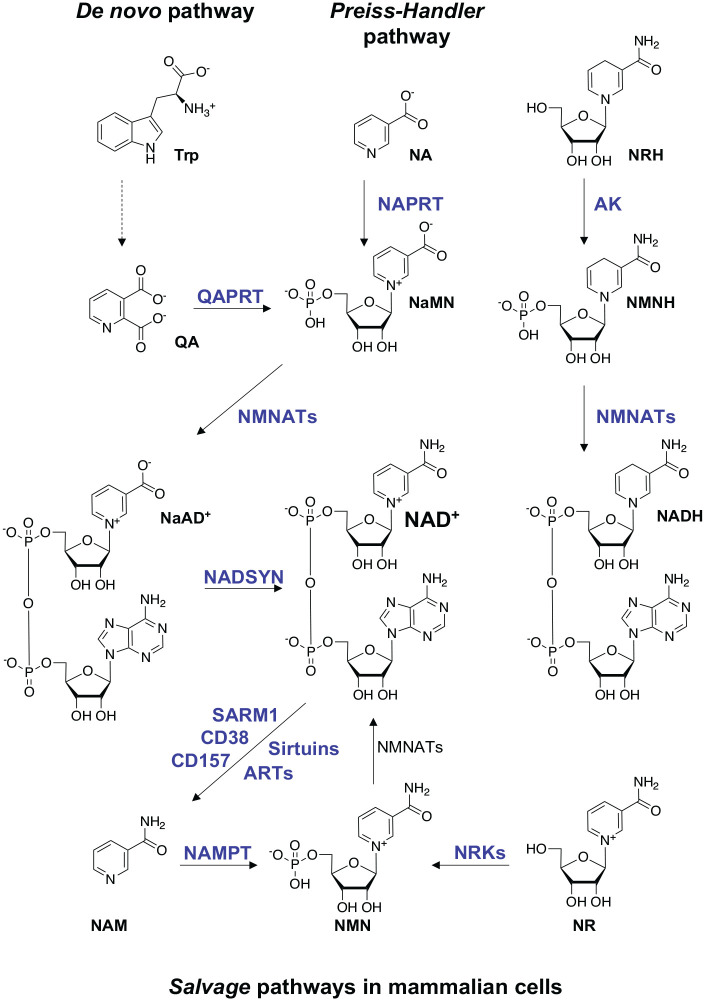
NAD^+^ biosynthetic pathways in mammalian cells. The Preiss-Handler pathway synthesizes NAD^+^ from dietary NA in three steps. NAPRT (nicotinate phosphoribosyltransferase) catalyzes the formation of NaMN (nicotinic acid mononucleotide) from NA and PRPP (5′-phosphoribosyl-1-pyrophosphate), which is converted into NaAD (nicotinic acid adenine dinucleotide) by NMNATs (nicotinamide mononucleotide adenylyltransferases). In the final step, NaAD is amidated into NAD^+^ by the NAD^+^ synthase (NADSYN1) enzyme. In the de novo pathway, dietary Trp is converted into QA (quinolinic acid) in a multistep reaction. QA is transformed into NaMN by QPRT (quinolinate phosphoribosyltransferase). NaMN, a common intermediate in the Preiss-Handler and de novo pathways, is converted into NaAD by NMNATs and finally into NAD^+^ by NADSYN1. NAD^+^ can also be synthesized from salvage of NAM generated as a by-product by NAD^+^-consuming enzymes (Figure 3). The NAMPT (nicotinamide phosphoribosyltransferase) enzyme catalyzes the formation of NMN (nicotinamide mononucleotide) from NAM and PRPP, which is converted into NAD^+^ by NMNATs. In addition, NR, which is transported into cells by nucleoside transporters, integrates into this salvage pathway via its conversion into NMN by NRK (nicotinamide ribose kinase) 1 and 2. Recently, NRH (dihydronicotinamide riboside) was discovered as a novel NAD^+^ precursor. NRH is phosphorylated by adenosine kinase, generating reduced NMN (i.e., NMNH), which in turn is adenylated to NADH by NMNAT (Yang and others 2020).

**Figure 3.**
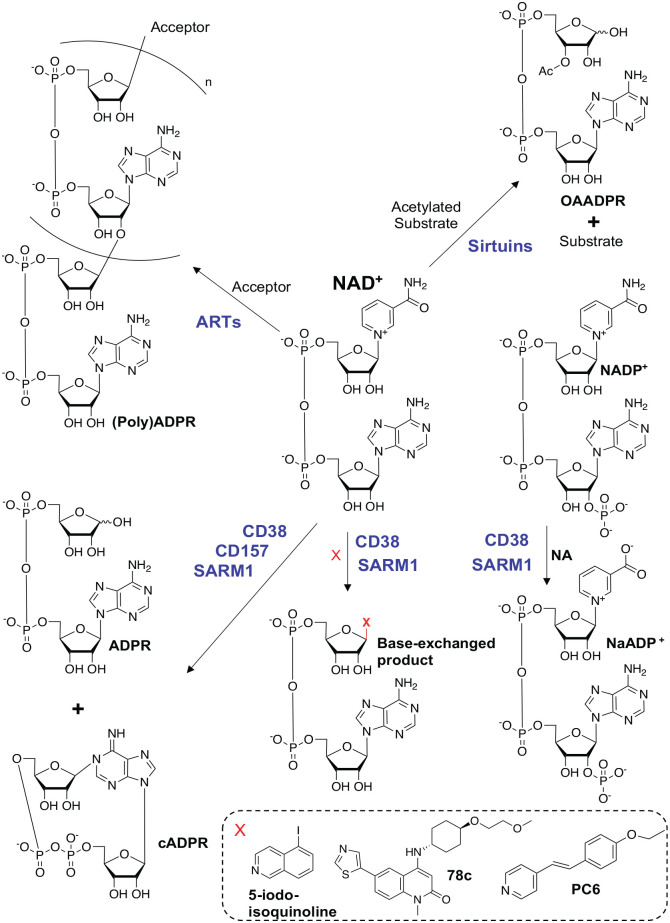
Nucleotide products produced by NAD^+^-consuming enzymes. The human ART family uses NAD^+^ as a substrate to generate NAM (nicotinamide) and mono- or polyADPR (adenosine diphosphate ribose) chains on proteins and nucleic acids. Only the linear polymer of ADPR is shown, although branching could occur every 40 to 50 ADPR units (Schreiber and others 2006). Sirtuins are NAD^+^-dependent deacetylases that transfer the acetyl group from protein substrates onto ADPR, producing OAADPR (O-acetyl-ADPR). CD38, CD157, and SARM1 are glycohydrolases that consume NAD^+^ to generate NAM, ADPR, and cADPR. CD38 and SARM1 can also catalyze base exchange reactions where the NAM moiety of NAD^+^ or NADP^+^ is replaced by various heterocyclic amines. 5-Iodo-isoquinoline and 78c are examples of prodrug bases that are substrates for SARM1- and CD38-catalyzed base exchange reactions, respectively. PC6 is a cell-permeant fluorescent probe specific for SARM1-catalyzed base exchange reactions.

The pyridine dinucleotide NAD^+^ and its phosphorylated version NADP^+^ are essential molecules in all three kingdoms of life and are involved in redox reactions by acting as hydrogen donors or acceptors (Berger and others 2004; Euler-Chelpin 1966; Warburg and Christian 1936). The NAD^+^/NADH couple is predominantly involved in oxidative reactions leading to the reduced version NADH, while the NADP^+^/NADPH couple mainly drives reductive reactions forming the oxidized version NADP^+^ (Canto and others 2015; Lautrup and others 2019; Yang and Sauve 2016). In mammals, NAD^+^ can be synthesized from four precursors: Trp (tryptophan) and the pyridine bases NA (nicotinic acid), NAM (nicotinamide), and NR (nicotinamide riboside) (Beadle and others 1947; Bender 1983; Bieganowski and Brenner 2004; Bogan and Brenner 2008; Preiss and Handler 1958; Raffaelli and others 2002; Revollo and others 2004) (Figure 2). Although NAD^+^ can be synthesized de novo from Trp, the main source of NAD^+^ in mammalian cells is from salvage via NAM. In addition to its role in redox reactions, NAD^+^ is used as a substrate by several enzyme families: ARTs (ADP-ribosyl transferases) (Luscher and others 2018; Miwa and others 1974; Miwa and Sugimura 1971) and sirtuins (Imai and others 2000), as well as the NAD^+^ glycohydrolases CD38 (Howard and others 1993), CD157 (Hirata and others 1994), and SARM1 (Essuman and others 2017) (Figure 3).

### NMNAT2

**Figure 4.**
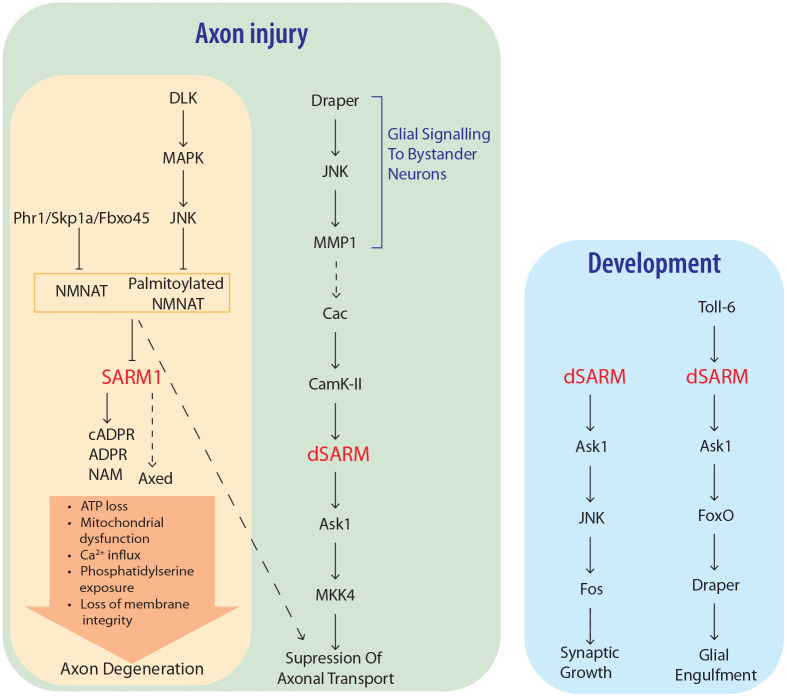
Up- and downstream effectors of SARM1 function in divergent pathways to regulate axon degeneration and development. Axonal injury results in loss of NMNAT via turnover by MAPK and ubiquitin ligase pathways, leading to SARM1 NADase and base exchange activities. Downstream events culminating in axon degeneration include Axed activation, ATP loss, loss of mitochondrial motility and depolarization, calcium influx, phosphatidylserine exposure on the cell membrane, and loss of membrane permeability. Ask1 acts downstream of SARM1 to regulate suppression of axonal transport after injury, as well as in developmental contexts regulating synaptic growth and clearance of debris by glia.

In all the biosynthetic routes, NAD^+^ or NaAD are ultimately produced by the transfer of the adenylyl moiety of ATP to NMN by NMNAT (Figure 2). Three isoforms of NMNAT exist in mammalian cells, and they exhibit different catalytic properties, quaternary structure, intracellular localization, and tissue distribution (Fortunato and others 2022). The least efficient isoform, NMNAT2 (Sorci and others 2007), is expressed predominantly in the brain and is associated with the cytosolic side of the Golgi apparatus and Golgi-derived vesicles (Lau and others 2010; Mayer and others 2010). It is the most labile NMNAT isoform and is rapidly degraded upon axonal injury (Gilley and Coleman 2010). Importantly, NMNAT2 is an axonal survival factor, as it is the only isoform whose depletion triggers spontaneous axon degeneration in cultured mouse neurites in vitro (Gilley and Coleman 2010). Axon degeneration caused by NMNAT2 loss can be counteracted by overexpressing NMNAT isoforms or the WLD^S^ (Wallerian degeneration slow) mutant (described later), demonstrating its neuroprotective role (Coleman and Hoke 2020; Gilley and Coleman 2010; Sasaki and others 2009; Yahata and others 2009; Yan and others 2010). Subcellular localization of NMNAT2 is determined by palmitoylation, with modified and nonmodified NMNAT2 forming two distinct pools that are independently targeted for degradation (Summers and others 2018) (Figure 4). Palmitoylation facilitates association of NMNAT2 with vesicle membranes, likely for anterograde transport from the Golgi to the axon (Milde and others 2013). Membrane-associated NMNAT2 is targeted for degradation by the MAP3Ks (mitogen-activated protein kinase kinase kinase), DLK (dual leucine zipper kinase) and LZK (leucine zipper kinase), and disrupting this association increases the stability of NMNAT2 (Summers and others 2018). A smaller, nonpalmitylated subpopulation of NMNAT2 is localized to the cytosol and is separately targeted for degradation by an atypical Skp1a-Phr1-Fbxo45 ubiquitin ligase complex (Babetto and others 2013; Xiong and others 2012; Yamagishi and Tessier-Lavigne 2016). Inhibiting either mechanism of NMNAT2 turnover is axoprotective; however, dual inhibition provides synergistic, robust axonal protection (Summers and others 2018).

### WLD^S^

Initial understanding of Wallerian degeneration assumed that it was a passive process, wherein the axon degeneration progressed away from the site of injury, following the gradual depletion of a hypothesized trophic factor essential to axon maintenance (Lubińska 1977, 1982). However, the discovery of a strain of C57BL/6/Ola mice, in which degeneration was significantly delayed, revealed that injury-induced degeneration is a genetically regulated program (Lunn and others 1989; Perry and others 1990). The WLD^S^ phenotype precipitated much of what is now understood about Wallerian degeneration. This phenotype was found to be attributable to a fusion gene encoding a chimeric protein consisting of the N-terminal 70 amino acids of UBE4B (ubiquitination factor E4B), NMNAT1, and an 18-residue linker from the untranslated region of NMNAT1 connecting the two domains (Conforti and others 2000; Lyon and others 1993; Mack and others 2001). The nuclear localization of NMNAT1 led to the initial idea that WLD^S^ may confer protection via genetic regulation by the NAD^+^-dependent histone deacetylase sirtuin 1 (Araki and others 2004; Magni and others 2004). However, it was found that the protective effects of WLD^S^ were due to the redistribution of NMNAT1 into the axons through interactions between the molecular chaperone VCP (valosin-containing protein) and the N-terminal residues, indicating a local mechanism of protection in the axon (Avery and others 2009; Beirowski and others 2009; Wang and others 2005). Specifically, translocation of NMNAT1 to the axons and synapses was found to mediate protection of the axon after injury by substituting for the natural turnover of the more labile, cytosolic isoform NMNAT2 (Babetto and others 2010; Gilley and Coleman 2010). NMNAT2 was therefore identified as the essential trophic factor originally hypothesized three decades prior (Gilley and Coleman 2010). Since its discovery, the WLD^S^ mutant has shown axonal protection and increased neural survival and function in several models of central, peripheral, and retinal neurodegenerative disorders (Krauss and others 2020).

### SARM1

**Figure 5.**
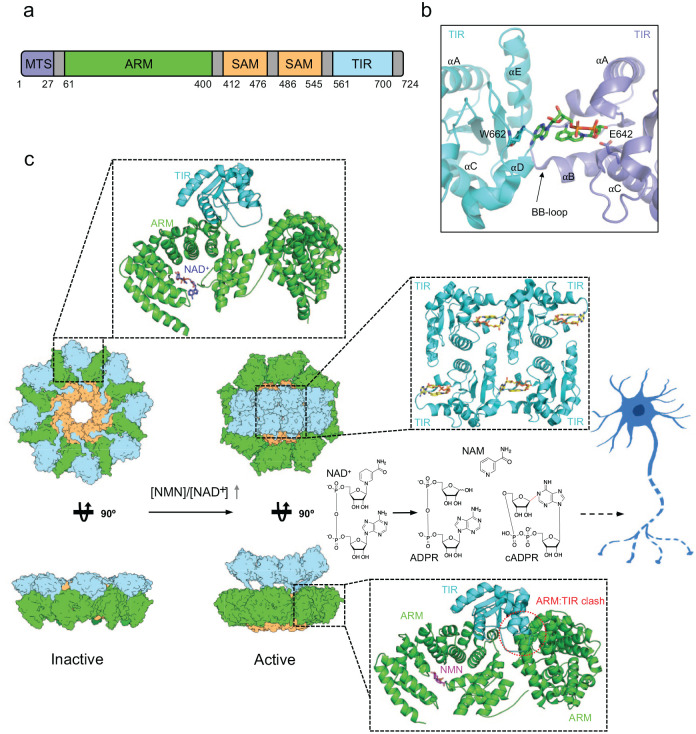
Structural basis of SARM1 regulation and activation. (A) Schematic representation of protein domains and motifs found in SARM1. (B) Crystal structure of SARM1 TIR domain in complex with the NAD^+^ mimetic 8-amino-isoquinoline adenine dinucleotide (green sticks; PDB: 7NAH). (C) SARM1 activation mechanism. Left panel: cryo-EM structure of the inactive state of SARM1 (PDB: 7ANW). The inset shows the interaction between the ARM and TIR domains. NAD^+^ is highlighted in purple. Right panel: cryo-EM structure of the active state of SARM1 in complex with NMN (PDB: 7NAL and 7NAK). The bottom inset highlights the ARM:TIR steric clash caused by reorientation of the ARM domain upon NMN binding. NMN is highlighted in magenta. The top inset shows the interactions between TIR domains and the inhibitor 1AD (yellow sticks).

SARM1 is a multidomain protein (Figure 5A) peripherally associated with mitochondria (Gerdts and others 2013; Panneerselvam and others 2012) and is primarily expressed in the brain with lower detectable levels observed in macrophages (Doran and others 2021). It was first described in 2001 as a conserved protein of unknown function in humans, mice, the fruit fly *Drosophila melanogaster*, and the roundworm *Caenorhabditis elegans* (Mink and others 2001). Because it contained a unique combination of sterile alpha (SAM) and armadillo repeat (ARM) motifs, it was named SARM1. It was subsequently annotated to have a C-terminal TIR (Toll/interleukin 1 receptor) domain, and due to the role of TIR domains in innate immune responses, it was presumed to be an adaptor protein involved in TLR (Toll-like receptor) signaling pathways (O’Neill and others 2003). Early research therefore focused on the role of SARM1 in innate immune pathways downstream of TLRs, which are an important class of receptors in the innate immune system that recognize pathogen-associated molecular patterns such as microbial lipids, lipoproteins, and nucleic acids and activate transcription factors such as NF-κB (nuclear factor–κB), AP-1 (activator protein 1), IRF-3 (interferon regulatory factor 3), as well as MAPK pathways, resulting in the production of proinflammatory cytokines and interferons (Ve and others 2012). Overexpression of human SARM1 in mammalian cells was not sufficient to induce expression of NF-κB or IRF3 (Liberati and others 2004); instead, SARM1 was shown to antagonize TLR pathways dependent on the signaling adaptors TRIF (TIR domain-containing adaptor-inducing interferon β) and MyD88 (myeloid differentiation factor 88) via TIR:TIR interactions (Carlsson and others 2016; Carty and others 2006; Peng and others 2010). SARM1 has also more recently been shown to regulate inflammasome formation (Carty and others 2019).

## SARM1 Is a Pro-neurodegenerative NADase

The functions of SARM1 extend beyond the innate immune system. SARM1 is predominantly expressed in neurons, and the search for a loss-of-function mutation that would phenocopy WLD^S^, proving Wallerian degeneration as an active process, led to the discovery that SARM1 was necessary for postinjury axon degeneration (Gerdts and others 2013; Osterloh and others 2012). Osterloh and others showed that genetic deletion of the *Drosophila* SARM1 orthologue (dSARM) preserved cut axons for >50 days. Loss of SARM1 was subsequently shown to also protect mouse neurons after injury (Gerdts and others 2013; Osterloh and others 2012). Expression of wild type dSARM or SARM1 in these mutants rescued the axon degeneration phenotype, demonstrating an essential role for dSARM/SARM1 in the degeneration process.

SARM1-induced axon degeneration involves rapid loss of axonal NAD^+^, resulting in a metabolic crisis ultimately leading to axon fragmentation (Gerdts and others 2015). Facilitating self-association of the SARM1 TIR domains have been shown to be necessary and sufficient to trigger this breakdown of NAD^+^ and can trigger axon degeneration, even in the absence of injury. The TIR domain of SARM1 has structural similarities to bacterial enzymes that hydrolyze CMP (cytosine monophosphate) into cytosine and ribose 5-phosphate (Horsefield and others 2019), and in 2017 Essuman and others demonstrated that the SARM1 TIR domain has NAD^+^ cleavage activity. This was a surprising discovery, as TIR domains had until then been reported to have only scaffolding functions in innate immunity signaling pathways (Nimma and others 2017). Mutation of a key catalytic glutamate residue (E642) in the TIR domain prevents axon degeneration after traumatic injury and vincristine-induced neurotoxicity, demonstrating that this intrinsic NADase activity of SARM1 is necessary to promote axon degeneration (Essuman and others 2017). The NADase activities of SARM1 require TIR domain self-association facilitated by the central SAM domains, which assemble into an octameric ring structure (Figure 5) (Horsefield and others 2019; Sporny and others 2019). SARM1 can also use NADP^+^ as a substrate and can catalyze multiple reactions, including hydrolysis of NAD^+^ into NAM (nicotinamide) and ADPR (adenosine diphosphate ribose); cyclization of NAD^+^ into cADPR (cyclic ADPR), which serves as a biomarker of SARM1 activity in neurons; and base exchange of the NAM moiety of NAD^+^ and NADP^+^ with various heterocyclic amines, including NA, vacor, 3-acetylpyridine, PC6, and prodrugs (Figure 3) (Angeletti and others 2022; Bratkowski and others 2022; WH Li and others 2021; Sasaki and others 2020; Shi and others 2022; Wu and others 2021; Zhao and others 2019).

In *Drosophila* the SARM1 NADase activity is required for development of the neuromuscular junction (NMJ) and TLR-mediated developmental glial phagocytosis via a pathway that involves the MAP3K Ask1 (Brace and others 2022; Herrmann and others 2022) (Figure 4). This pathway cannot be blocked by overexpression of NMNAT, and since Ask1 does not appear to be involved in SARM1-dependent axon degeneration, the mechanisms upstream and downstream of SARM1 in developmental signaling are most likely distinct to SARM1-dependent axon degeneration (Brace and others 2022; Herrmann and others 2022). Interestingly, while SAM domain–mediated oligomerization of SARM1 is important for axon degeneration, it is dispensable for developmental signaling (Herrmann and others 2022). Future work is required to understand how SARM1 is activated in developmental signaling and how SARM1 triggers activation of the Ask1 kinase.

## SARM1 Is a Metabolic Sensor of Axonal NAD^+^, NMN, and NaMN levels

The link between the protective role of NMNAT2 and prodegenerative SARM1 was established in a series of genetic experiments showing that axon degeneration induced by loss of NMNAT2 was dependent on SARM1. SARM1 knockout provided robust protection independent of NMNAT2 turnover in SCG (superior cervical ganglion) neurites and prevented degeneration in Nmnat2 siRNA knockdown neurons, indicating that SARM1 functions downstream of NMNAT2 loss (Gilley and others 2015). Furthermore, SARM1 deletion provided significantly increased protection against perinatal lethality caused by NMNAT2 deficiency when compared with WLDs, emphasizing the key regulatory role of SARM1 in axon degeneration induced by NMNAT2 loss (Gilley and others 2015; Gilley and others 2017). SARM1 NADase activity is also inhibited by expression of NMNAT, and it was therefore suggested that the depletion of NMNAT2 after injury activates SARM1 (Gerdts and others 2015; Gilley and others 2015; Gilley and others 2017; Sasaki and others 2016). However, the precise mechanism of SARM1 activation was a matter of debate for many years. Apparently conflicting results suggested that the causative factor for SARM1 activation was either the accumulation of the NMNAT2 substrate NMN, which was found to be prodegenerative, or the depletion of the product NAD^+^ as a result of NMNAT2 loss, with SARM1 NADase activity acting in a positive feedback loop, driving rapid NAD^+^ and energy depletion (Gerdts and others 2016).

Axons can be protected from injury-induced degeneration by preventing NAD^+^ depletion, either by exogenous application of NAD^+^ or by WLD^S^ (Araki and others 2004; Sasaki and others 2006; Sasaki and others 2009; Wang and others 2005). Rapid NAD^+^ depletion is also sufficient for spontaneous axon degeneration, giving a convincing argument for NAD^+^ depletion being the causative event for activation of the downstream Wallerian degeneration pathway (Gerdts and others 2015).

An alternative culprit was identified in the NAD^+^ precursor NMN, which accumulates in the axon after injury as NMNAT2 depletes (Di Stefano and others 2015). Preventing NMN accumulation by inhibition of NAMPT provides robust protection, despite lowering NAD^+^, and this protection can be overcome by the addition of exogenous NMN, which is able to induce degeneration in the absence of injury (Di Stefano and others 2015; Gilley and others 2015). Scavenging of NMN with NMN deamidase is also able to rescue axonal defects in NMNAT2 knockout mice (Di Stefano and others 2017). Additionally, a cell-permanent mimetic of NMN, CZ-48, activates SARM1 enzymatic activity by inducing TIR self-association (Zhao and others 2019). However, neither of these models explains why baseline changes to either metabolite are insufficient to drive injury-induced degeneration (Sasaki and others 2016).

This apparent conflict was reconciled with the discovery that SARM1 is a metabolic sensor of NMN/NAD^+^ ratio in neurons (Figley and others 2021). Treating axons with the NMN precursor NR was shown to activate SARM1 enzymatic activity but not lead to spontaneous degeneration. Instead, the effects of NR application were found to be context dependent. If injury occurred directly after NR treatment, when NMN levels were elevated but before the corresponding increase in NAD^+^, axons degenerated. However, after 24 h, axons were protected from injury, despite elevated levels of both metabolites, indicating that the activation of SARM1 is not dependent on individual changes in metabolites but on fluctuations in the ratio between NMN and NAD^+^. By independently altering the NMN or NAD^+^ levels in cultured primary neurons, Figley and others (2021) found that manipulations that either elevated NMN or decreased NAD^+^ levels can equally trigger SARM1 NADase activity. Consistent with these results, NMN and NAD^+^ were shown to activate and inhibit the NADase activity of recombinantly produced SARM1, respectively, and compete for binding to the same allosteric pocket in the ARM domain (Angeletti and others 2022; Bratkowski and others 2020; Figley and others 2021; Jiang and others 2020; Sporny and others 2020; Zhao and others 2019). The NMN/NAD^+^ ratio concept explains how SARM1 is controlled by NMNAT2. In healthy axons NMNAT2 converts NMN to NAD^+^, resulting in a low NMN/NAD^+^ ratio, which favors SARM1 inhibition. However, in injured or diseased neurons, axonal NMNAT2 transport is disrupted, leading to reduced NAD^+^ synthesis, accumulation of NMN, and thereby a higher NMN/NAD^+^ ratio, which favors SARM1 activation. While the physiologic ratio of axonal NAD^+^ and NMN has not been determined, the level of NAD^+^ is orders of magnitude higher than NMN in SGC neurites and DRG (dorsal root ganglia) neurons (Di Stefano and others 2015; Liu and others 2018). Steady state levels of NAD^+^ and NMN in the brain are approximately 300 and 6 µM respectively, and regulation of SARM1 activity at these levels supports a model of competitive binding to an allosteric regulatory site (Angeletti and others 2022; Mori and others 2014).

That SARM1 acts as a sensor of NMN/NAD^+^ ratio explains why inhibition of the NMN synthesizing enzyme NAMPT by small molecule inhibitors, such as FK866, provides short-term protection of axons from degeneration, but it does not fully explain why expression of a bacterial NMN deamidase, which converts NMN into NaMN, or cotreatment of neurons with FK866 and the NaMN precursor NaR (nicotinic acid riboside) provides significantly longer axonal protection than FK866 treatment alone (Di Stefano and others 2015; Liu and others 2018; Sasaki and others 2009). Recently, this mystery was solved by Sasaki and others (2021), who demonstrated that NaMN is a SARM1 inhibitor that competes with NMN for binding to the allosteric pocket in the ARM domain. NaMN is a much weaker SARM1 binder than NMN, and NaMN-mediated axonal protection is observed only when NMN levels are artificially lowered. It is therefore not clear under what circumstances NaMN might be relevant for axonal protection in untreated neurons.

## Downstream Effectors of SARM-Dependent Axon Degeneration

The approximate events that follow injury-dependent activation of SARM1 are known, with initial SARM1-dependent loss of cellular ATP preceding a failure of mitochondrial motility and membrane potential (Figure 4). Later events involve calcium influx and exposure of phosphatidylserine, which is shortly followed by axonal fragmentation (Ko and others 2021). However, the precise molecular mechanisms controlling these events remain an area requiring investigation.

Late-stage calcium influx is SARM1 dependent, and it is suggested that this is largely from extracellular sources such as L-type Ca^2+^ channels, as blocking these channels conveys greater protection than inhibiting calcium release from the endoplasmic reticulum and mitochondria (Ko and others 2021; Loreto and others 2015). However, this does not exclude a contributing role for intracellular calcium channels in degeneration, and knockdown of the endoplasmic reticulum calcium channel RyR (ryanodine receptor) inhibits paclitaxel-induced axon degeneration (Y Li and others 2021).

Given the known roles of ADPR and cADPR as calcium mobilizers targeting the TRPM2 (transient receptor potential cation channel subfamily M member 2) and RyR calcium channels (Galione and others 1991; Perraud and others 2001; Sano and others 2001), they have been investigated as the culprits of SARM1-dependent calcium influx. SARM1 activity is the main regulator of cellular cADPR levels, and while cADPR antagonists are protective in a paclitaxel model of degeneration, it does not appear to be the central regulator of calcium influx in axotomy-induced degeneration (Y Li and others 2021; Sasaki and others 2020).

After axotomy, calpastatin, a suicide inhibitor of the protease calpain, is depleted. This occurs downstream of SARM1 and is likely due to elevation of cellular calcium, which activates calpain and causes the degradation of calpastatin (Yang and others 2013). Once inhibition by calpastatin is released, calpains are responsible for the degeneration of the axonal cytoskeleton (Ma and others 2013). Chelation of extracellular calcium and inhibitors of calpains prevent morphologic degeneration of axons but not electrophysiologic function, and calcium influx and calpain activation likely represent the final steps in fragmentation of a metabolically dead axon.

The role of MAPKs in axon degeneration has long been known, with inhibition of a MAPK cascade including DLK and JNK providing protection after axonal injury comparable to that of SARM1 knockout (Yang and others 2015). However, recent research shows that the relationship between MAPK signaling and SARM1 activity may be more complicated than initially appreciated (Figure 4). Upstream of SARM1, the DLK signaling cascade promotes degeneration by regulating the turnover of NMNAT2 (Walker and others 2017). The relevance of MAPKs for degeneration downstream of SARM1 has long been debated, but recent work in *Drosophila* suggests that they may function in alternative signaling roles. Hsu and others (2021) showed that dSARM NADase activity as well as a cacophony (Cac)/dSARM/MAPK signaling cascade is required for an initial phase of axon transport blockage after axotomy. Interestingly, NMNATs may be required for transport blockage, suggesting additional postaxotomy functions for NMNATs. Axon blockage also occurs in bystander neurons, and this requires signaling to glia through Draper. Draper/Ask1 signaling downstream of dSARM plays a role in development, and in *C. elegans* this pathway appears to regulate regeneration of axons (Brace and others 2022; Herrmann and others 2022; Julian and Byrne 2020). These diverging roles show multifaceted functions for SARM1 in neurons and will be an important future area of research.

Another known downstream effector of SARM1 is the *Drosophila* protein Axed. Knockout of Axed is able to confer complete protection of axons from injury-induced degeneration in the face of activated dSARM and depletion of NMNATs, a particularly remarkable phenotype considering that unlike mammals, NMNAT is the sole NAD^+^ synthase in *Drosophila* (Neukomm and others 2017). Genetic manipulation of Axed is additionally the most downstream point in the Wallerian degeneration pathway where long-term axon functionality is conserved, and identification of a mammalian homolog or analogous mechanism may represent an ideal target for therapeutic intervention.

## Mechanisms of SARM1 Regulation, Activation, and NAD^+^ Cleavage—Insights from Structural Biology

As discussed earlier, SARM1 is a multidomain protein, and early structure function studies showed that the central tandem SAM domains are required for oligomerization, while the N-terminal ARM domain is autoinhibitory (Gerdts and others 2013; Horsefield and others 2019; Sporny and others 2019). Several recent crystal and cryo-EM studies have revealed the architecture of full-length SARM1 and provided detailed insight into how SARM1 is regulated and activated and how it cleaves NAD^+^ (Bratkowski and others 2020; Figley and others 2021; Jiang and others 2020; WH Li and others 2021; Shen and others 2021; Shi and others 2022; Sporny and others 2020) (Figure 5). Crystal structures of the SARM1 TIR domain in complex with inhibitors and NAD^+^ mimetics (Figure 5B) showed that the active site spans two TIR-domain molecules, explaining the requirement of TIR domain self-association for NADase activity (Bratkowski and others 2022; Shi and others 2022). Full-length SARM1 forms an octameric ring complex, mediated by SAM domain interactions, and this oligomeric state is required for SARM1’s degenerative capability. The ARM domains form an outer ring, interacting with the SAM domains. In the inactive complex, the TIR domains bind the intrachain and neighboring ARM domains, maintaining a distance of 25 Å between TIRs (Figure 5B). The ARM:TIR interactions regulate SARM1 by preventing TIR self-association, and disruption of the ARM:TIR interface by site-directed mutagenesis produces a constitutively active SARM1 (Bratkowski and others 2020). In cryo-EM structures of human SARM1 and crystal structures of *Drosophila* SARM1, the ARM domain revealed an unusual compact superhelix with the N- and C-terminal regions collapsed around the allosteric activator NMN, or the allosteric inhibitors NAD^+^ and NaMN (Figure 5C) (Figley and others 2021; Jiang and others 2020; Sasaki and others 2021; Sporny and others 2020). In two recent cryo-EM structures of hSARM1 incubated with NMN, the ARM domains have undergone a significant reorientation with respect to the octameric SAM domain ring (Hou and others 2022; Shi and others 2022). In both structures, NMN interacts with the loop between residues 310 and 325, causing the ARM7 and ARM8 repeats to move toward NMN, resulting in a compaction of the ARM domain. In the structure by Shi and others, there is no visible density for the TIR domain, and the ARM domain has rotated upward in a clockwise fashion. Structural comparisons with inactive state structures suggest that this NMN-induced rotation leads to a collision with the neighboring TIR domain, resulting in destabilization of the ARM:TIR interaction (Figure 5C). By contrast, the NMN bound structure reported by Hou and others still has TIR domains attached to the ARM domains. However, this structure is stabilized by a SAM and ARM domain binding nanobody, and consequently the ARM domain adopts a completely different conformation that does not collide with neighboring TIR domains. Hence, it is likely that this structure corresponds to a nanobody-trapped intermediate state of the SARM1 activation process.

Overall, the structural data on SARM1 suggest the following activation mechanism. In an uninjured axon, the SARM1 octamer is inactive, with the TIR domains separated by the regulatory ARM domains bound to NAD^+^. After injury, turnover of NMNAT2 and loss of NAD^+^ synthase activity result in a large shift in the metabolic ratio of NMN and NAD^+^. Accumulated NMN then binds to the SARM1 ARM domain in place of NAD^+^, resulting in 1) compaction of the ARM domains; 2) reorientation of the ARM domains causing a clash with the neighboring TIR domains; 3) release of the TIR domains; and 4) self-association of the TIR domains into an open-ended assembly with up to six catalytically competent active sites per SARM1 octamer, resulting in rapid loss of NAD^+^ and axon degeneration.

## SARM1 in Neurodegenerative Diseases, Environmental Neurotoxicity, and Viral Infection

In recent years, there has been emerging evidence suggesting a contributory role of SARM1 in many neurodegenerative disorders (Table 1), environmental neurotoxicity, and viral infections.

**Table 1.** Effects of SARM1 Deletion on Animal-Based Disease Models.

Diseases	Insults	Models	SARM1 KO Effects	References
Peripheral nerve injury	Vincristine	Mouse	Blocks the development of CIPN	Geisler and others (2016)
	Paclitaxel	Mouse	Partially protected from the thermal sensitivity and epidermal innervation; no effect on the decrease of tail SNAP amplitudes	Turkiew and others (2017)
	Cisplatin	Mouse	Resistant to the development of distal axonal sensory neuropathy	Cetinkaya-Fisgin and others (2020)
	High-fat diet	Mouse	Resistant to the development of prediabetes, thermal hypoalgesia, as well as the decrease of tail SNAP amplitudes	Turkiew and others (2017)
Traumatic brain injury	Impact on the closed skull	Mouse	Protected from neuroinflammation, corpus callosum atrophy, axon damage, myelin loss; improve motor learning and normalize time spent sleeping	Bradshaw and others (2021), Henninger and others (2016), Marion and others (2019)
Glaucoma	TNF-α	Mouse	Resistant to the loss of axons and oligodendrocyte in optic nerves	Ko and others (2020)
Amyotrophic lateral sclerosis	Gain-of-function SARM1 mutations	Mouse	Expression of SARM1 variants in the *Sarm1*^−/−^ DRG neurons leads to axon degeneration and cell death	Bloom and others (2022)
	*SOD1*^G93A^ mutation	Mouse	Neither delays axon degeneration nor decelerates disease progression	Peters and others (2018)
Charcot-Marie-Tooth disease type 2A	*Mfn2*^H361Y^ mutation	Rat	Protected from axonal, synaptic, and muscle defects, as well as mitochondrial defects	Sato-Yamada and others (2022)

CIPN, chemotherapy-induced peripheral neuropathy; DRG, dorsal root ganglion; KO, knockout; MFN2, mitofusin 2; SNAP, sensory nerve action potential; SOD1, superoxide dismutase 1.

### SARM1 in Neurodegenerative Diseases

Peripheral neuropathy is the most common neurodegenerative disorder, characterized by weakness, numbness, and pain due to nerve damage, which can be hereditary or acquired (DiAntonio 2019). It is one of the most common side effects of chemotherapy agents, referred to as CIPN (chemotherapy-induced peripheral neuropathy). Many such neuropathies feature dying back of axons through unknown mechanisms (Fukuda and others 2017). However, a study of CIPN induced in a mouse model using vincristine, an anticancer agent, demonstrated that loss of SARM1 confers a protective effect against axon neuropathies (Geisler and others 2016). Similarly, protection has been seen in mouse models of paclitaxel- and cisplatin-induced CIPN, in which *Sarm1*^-/-^ mice were resistant to peripheral neuropathies that occurred in wild-type mice (Cetinkaya-Fisgin and others 2020; Y Li and others 2021; Turkiew and others 2017). In both cases, calpain seems to be required for SARM1-dependent neuropathy.

TBI (traumatic brain injury) often causes axon damage in white matter tracts and increases the risk of neurodegeneration (Bradshaw and others 2021). Emerging evidence suggests that SARM1 plays an important role in the prodegenerative pathway of TBI, because the loss of SARM1 protects mice from various pathogenic phenotypes. For example, *Sarm1*^-/-^ mice develop less β-amyloid precursor protein aggregates in the corpus callosum (Henninger and others 2016) and have attenuated TBI-induced myelin loss, white matter atrophy, and neuroinflammation as compared with wild-type mice (Bradshaw and others 2021; Marion and others 2019).

Glaucoma is a group of eye diseases leading to irreversible visual impairment due to the continuous degeneration of RGC (retinal ganglion cell) axons (Weinreb and others 2014). Neuroinflammation is the main cause of glaucoma. Using a neuroinflammatory mouse model of glaucoma induced by the injection of TNF-α (tumor necrosis factor α), Ko and others (2020) showed that SARM1 is required for the loss of RGC axons and oligodendrocytes in optic nerves and axon degeneration in sensory neurons. SARM1-induced axon degeneration in sensory neurons involves a noncanonical necroptotic signaling mechanism in which the MLKL (mixed lineage kinase domain–like) pseudokinase, usually acting as the final executioner in the canonical necroptosis pathway, induces loss of NMNAT2 and STMN2 (stathmin 2, also known as SCG10) to initiate SARM1-dependent axon degeneration (Ko and others 2020).

Recent studies have identified the presence of SARM1 variants with constitutively hyperactive NADase activity in patients with ALS and other motor nerve disorders, demonstrating the role of SARM1 as a candidate genetic risk factor for these diseases (Bloom and others 2022; Gilley and others 2021). These SARM1 variants mostly involve missense mutations (e.g., V184G, L223P) and small in-frame deletions (e.g., Δ226-232) in the autoinhibitory ARM domain and exhibit similar NADase activities to a fully active fragment of SARM1 consisting of the SAM and TIR domains (SARM1^SAM-TIR^). Expression of patient-specific hyperactive SARM1 variants in cultured SCG neurons (Δ229-235 as a representative variant) led to the death of neurons. In cultured DRG neurons, the V184G mutant induced axon degeneration, cell death, and neuroinflammation, further supporting involvement of the SARM1 prodegenerative pathway. However, loss of SARM1 seems not to protect motor neurons from degeneration in a *SOD1*^G93A^ (SOD1: superoxide dismutase 1) mouse model of ALS, suggesting that neurodegeneration in this particular model of ALS involves a SARM1-independent pathway (Peters and others 2018).

SARM1 has been shown to play a prodegenerative role in axonopathies characterized by mitochondrial dysfunction, such as CMT (Charcot-Marie-Tooth) disease type 2A caused by mutations in the *MFN2* (mitofusin 2) gene (Loreto and others 2020; Sato-Yamada and others 2022). Using a rat model carrying the strong pathogenic mutation *Mfn2*^*H361Y*^ to model the CMT condition, Sato-Yamada and others (2022) showed that knockout of SARM1 in these rats suppresses axon degeneration, NMJ defects, and muscle atrophy, as well as mitochondrial impairment. Because mitochondrial dysfunction has been shown to activate SARM1-dependent axon degeneration through the deletion of NMNAT2, the underlying mechanism may involve a pathway regulated via a mitochondrial-SARM1 feedback loop.

### SARM1 in Environmental Neurotoxicity

Two recent studies have provided evidence that SARM1 is an essential mediator of environmental neurotoxicity because it can be targeted and activated by neurotoxins to induce axon degeneration (Loreto and others 2021; Wu and others 2021). In a study of the mechanism of action of the pyridine derivative vacor, a banned rodenticide and powerful neurotoxin associated with human nervous system disorders, Loreto and others (2021) showed that vacor neurotoxicity is predominantly SARM1 dependent and the *Sarm1*^-/-^ neurons and axons are entirely resistant to death upon vacor treatment. It was further demonstrated that the vacor metabolite VMN (vacor mononucleotide) is the actual activator of SARM1, mimicking NMN by inducing the compact, activated conformation of the autoinhibitory ARM domain. VMN interacts more strongly with the protein than NMN and activates SARM1 more efficiently. Interestingly, high concentrations of VMN inhibit the NADase activity of recombinant human SARM1, suggesting that there could be a second binding site, possibly the catalytic site in the TIR domain. Another study elucidated the mechanism of neurotoxicity for 3-AP (3-acetylpyridine), a nicotinamide analogue first reported in the 1940s (Wu and others 2021). Like VMN, 3-AP–induced axon degeneration and neuronal cell death are dependent on SARM1 activation, and this process relies on conversion of 3-AP to 3-APMN (3-acetylpyridine mononucleotide). Although the authors did not investigate the mechanism in detail, the structural similarity to NMN suggests that 3-APMN likely binds to the allosteric site in the ARM domain and activates the protein using a mechanism similar to NMN and VMN. Wu and others identified 2-aminopyridine as another SARM1-dependent neurotoxin. In summary, SARM1 can be selectively activated by pyridine analogues, which are widely used in drugs and industrial compounds, providing valuable information for the future use of related chemicals in pharmaceutical and environmental applications.

### SARM1 in Viral Infections

Neurotropic viruses can infect neurons, resulting in severe CNS diseases. Some invade the neurons from axon terminals and move along the axons to reach neuronal soma, where they can remain latent or propagate (Richards and others 2021; Salinas and others 2010). The newly synthesized virions are required to be transported back to the axons for further release to infect other neurons. To impede the viral spread, neurons are thought to have evolved a mechanism of rapid axon degeneration to clear the damaged and unhealthy neurons. Consistent with this notion, SARM1 has been shown to regulate axon degeneration during lyssavirus (Sundaramoorthy and others 2020) and Zika virus infections (Crawford and others 2022), although the downstream pathways may differ from injury-induced degeneration. Axon degeneration induced by lyssavirus is regulated by NAD^+^ loss and calpain activation (Sundaramoorthy and others 2020), while Crawford and others (2022) showed that NAD^+^ loss observed following Zika virus infection is independent of SARM1 NADase activity at 24 h postinfection. Other functions of SARM1, such as hydrolyzing NADP^+^ and performing base exchange reactions, as well as the immunity-related activities, may play a role in the early stages of infection. A role for SARM1 in WNV (West Nile virus) infections has also been reported. *Sarm1*^-/-^ mice exhibited decreased levels of TNF-α and increased viral replication and neuronal cell death after infection with WNV (Szretter and others 2009), and this increased susceptibility to WNV was recently confirmed in a CRISPR/Cas9-generated SARM1 null mouse that excluded contributions from any passenger mutations (Uccellini and others 2020). Because WNV activates TLR signaling, SARM1 may have an innate immunity role during WNV infection.

### Therapeutic Targeting of SARM1

Several sets of small-molecule inhibitors targeting SARM1 have been discovered and developed in the past 3 years, with their potency evolving from the micromolar range to the single-digit nanomolar level (Table 2). In 2020, two Zn^2+^-containing compounds were discovered as noncompetitive inhibitors of SARM1 NADase activity, possibly engaging one or more cysteine residues in the TIR domain with micromolar affinities independent of the substrate (Loring and others 2020). However, Zn^2+^ is known to interact with many other proteins and bind strongly to metallothionein in neurons (Colvin and others 2003). Thus, such Zn^2+^-containing compounds with modest potency are unlikely to be therapeutically useful. In early 2021, a series of isoquinoline derivatives was shown to have not only potent inhibition of NADase activity against SARM1^SAM-TIR^ but also cellular activities that protect axons from degeneration (Hughes and others 2021). These isoquinoline compounds were later demonstrated to undergo NAD^+^-dependent, SARM1-catalyzed base exchange reactions to generate the bona fide inhibitor at the active site (Shi and others 2022). More recently, a series of pyridine derivatives was shown to utilize the same base exchange mechanism to achieve potent inhibition of SARM1 and deliver safe and efficacious neuroprotection in preclinical models of neurologic injury and disease (Bratkowski and others 2022). This demonstrates that utilizing this base exchange mechanism could be a viable therapeutic strategy for SARM1 inhibition and neuroprotection. Several noncompetitive and competitive SARM1 inhibitors were also identified through a high-throughput screening campaign (Khazma and others 2022). Although these compounds showed strong protection against axon degeneration, the exact location of the binding sites was not identified.

**Table 2.** Representative SARM1 Inhibitors.

Reference	Compound	Potency	Mechanism	Assay
Loring and others (2020)	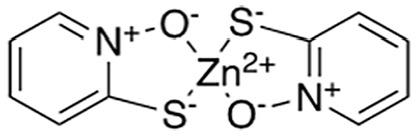 Zinc pyrithione **ZnCl_2_** Zinc chloride	IC_50_ 20 µM *K*_D_ 15 µM	Noncompetitive	SARM1 TIR domain lysates; etheno-ADPR detection by fluorescence
		IC_50_ 10 µM *K*_D_ 3.3 µM		
Hughes and others (2021)	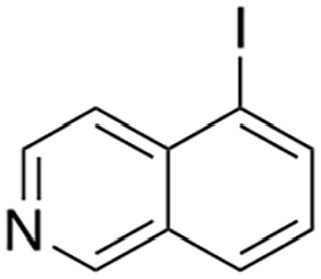 5-iodo-isoquinoline	IC_50_ 75 nM	Base exchange	SARM1^SAM-TIR^ lysate; ADPR detection by MS
	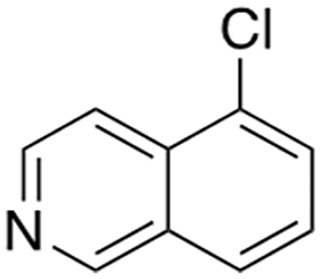 5-chloro-isoquinoline	IC_50_ 1.1 µM		
WH Li and others (2021)	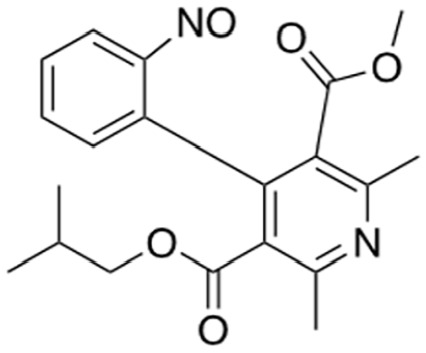 dHNN	IC_50_ 2.4 µM	Covalent; multiple possible cysteines	SARM1 with N-terminal mitochondrial localization signal truncated; detection of a pyridine styryl adenine dinucleotide derivative (PAD6) by fluorescence; ADPR detection by HPLC; cADPR detection in DRG neurons by the cycling assay (Graeff and Lee, 2002)
Bosanac and others (2021)	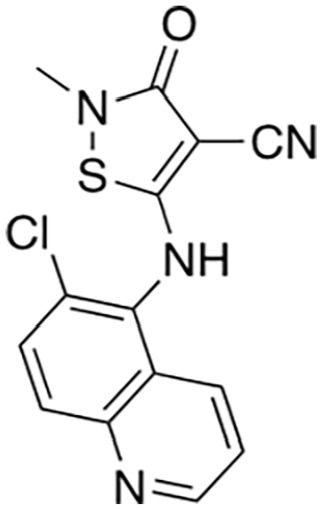 Isothiazole (9)	IC_50_ 0.16 µM	Irreversible; possibly C635	SARM1^SAM-TIR^ lysate; ADPR detection by MS
	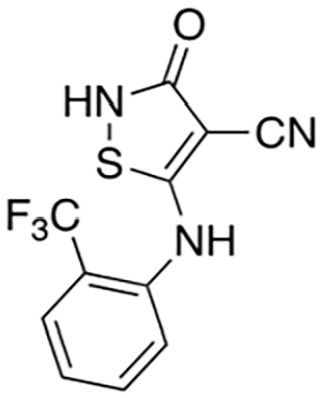 Isothiazole (4)	IC_50_ 0.37 µM		
Feldman and others (2022)	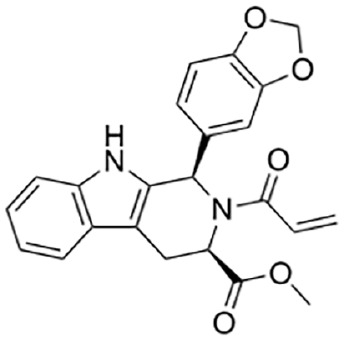 Tryptoline acrylamide EV-99	IC_50_ 4.7 µM	Covalent; selective at C311, near ARM allosteric site	SH-SY5Y cells treated in situ with inhibitor for 3 h, followed by 50-µM vacor for 4 h; cADPR detection by LC-MS
	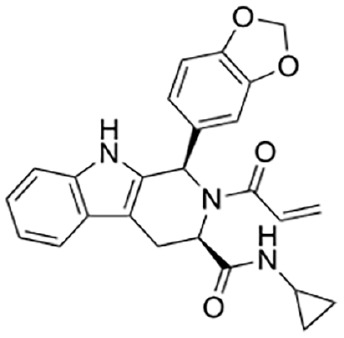 Tryptoline acrylamide WX-02-37	IC_50_ 1.5 µM		
Bratkowski and others (2022)	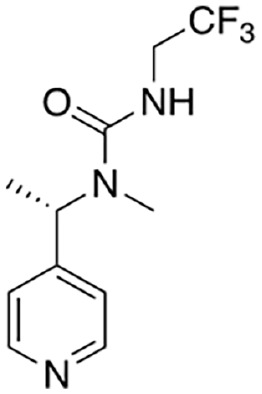 NB-3	IC_50_ 0.195 µM *K*_D_ 32.9 nM	Base exchange; uncompetitive	SARM1^SAM-TIR^ purified protein (with N-terminal MBP-tag); NAD^+^/NAM detection by HPLC
	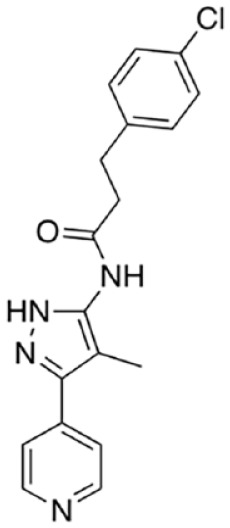 NB-7	IC_50_ 0.025 µM *K*_D_ 4.94 nM		SARM1^SAM-TIR^ purified protein (with N-terminal MBP-tag) protein; NAD^+^/NAM detection by HPLC
Khazma and others (2022)	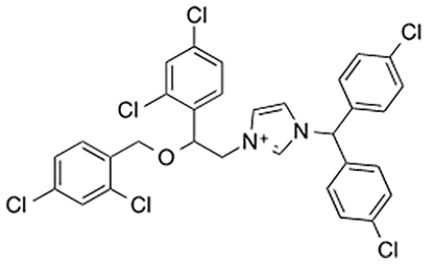 TK106	IC_50_ 10.8 µM	Noncompetitive	Wild type SARM1; ADPR detection by HPLC
	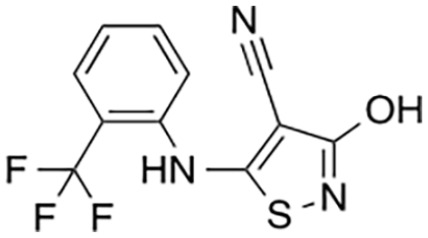 TK138	IC_50_ 2.9 µM	Competitive	

HPLC, high-performance liquid chromatography; LC, liquid chromatography; MS, mass spectrometry.

Covalent SARM1 inhibitors have recently been reported. A series of isothiazole-based compounds that may form enzyme-inhibitor adducts via C635 in the TIR domain showed irreversible inhibition of SARM1 NADase activity and were the first SARM1 inhibitors that exhibited axon protection in vivo (Bosanac and others 2021). A derivative of nisoldipine (NSDP) also showed irreversible inhibition of SARM1 NADase activity (WH Li and others 2021). This compound reacted with several cysteine residues, including C311, which is located near the allosteric site of the ARM domain. More recently, a series of highly selective covalent inhibitors has been reported to specifically target C311 (Feldman and others 2022). These tryptoline acrylamides were shown to prevent vincristine- and vacor-induced degeneration of rodent DRG neurons, but how they lead to SARM1 inhibition is not yet understood.

## Conclusions

SARM1 has in the last decade emerged as the central protein responsible for executing the axon degeneration program and has been the focus of intense research, leading to tremendous advances in understanding its structure, function, and regulation. SARM1 and NMNAT2 are the key focal points that regulate axon degeneration through manipulating and sensing changes in axonal NAD^+^ metabolism.

The signaling pathways that lead to axon degeneration after SARM1 activation remain poorly understood and will be one important area of future research. These downstream events clearly involve calcium fluxes, but the transporters responsible have not been identified. SARM1 also produces the calcium mobilizers cADPR and NaADP via its NADase and base exchange activities but their roles in axon degeneration are not well understood. The involvement of MAPKs and human orthologues of the *Drosophila* protein Axed in events downstream of SARM1 are other areas of further study.

There is urgent need for therapies for neurodegenerative diseases because most available drugs only treat the symptoms and slow disease progression. Axon degeneration is a pathologic feature of many neurologic disorders, and SARM1 variants have been found to be associated with ALS and other neurologic disorders. Considering that *Sarm1*^-/-^ mice appear healthy, SARM1 has become a popular drug target, and recent discoveries indicate that SARM1 is druggable by small molecules with diverse modes of action that include noncompetitive inhibition, uncompetitive inhibition/base exchange, and covalent inhibition. Given these rapid developments, we expect more SARM1 inhibitors with different scaffolds and modes of action as well as better pharmacokinetic profiles to be discovered soon and ideally progressed to the clinic.
